# Engineering cell fate: Applying synthetic biology to cellular reprogramming

**DOI:** 10.1016/j.coisb.2020.09.002

**Published:** 2020-09-21

**Authors:** Nathan B. Wang, Adam M. Beitz, Kate E. Galloway

**Affiliations:** Department of Chemical Engineering, MIT, 25 Ames St., Cambridge, MA, 02139, USA

**Keywords:** Cellular reprogramming, Synthetic biology, Epigenetics, Gene regulatory networks, Delivery, Latent donor identity, Signaling, Cell state, Cell fate, Gene circuits, Lineage tracing, Barcoding

## Abstract

Cellular reprogramming drives cells from one stable identity to a new cell fate. By generating a diversity of previously inaccessible cell types from diverse genetic backgrounds, cellular reprogramming is rapidly transforming how we study disease. However, low efficiency and limited maturity have limited the adoption of *in vitro*-derived cellular models. To overcome these limitations and improve mechanistic understanding of cellular reprogramming, a host of synthetic biology tools have been deployed. Recent synthetic biology approaches have advanced reprogramming by tackling three significant challenges to reprogramming: delivery of reprogramming factors, epigenetic roadblocks, and latent donor identity. In addition, emerging insight from the molecular systems biology of reprogramming reveal how systems-level drivers of reprogramming can be harnessed to further advance reprogramming technologies. Furthermore, recently developed synthetic biology tools offer new modes for engineering cell fate.

## Introduction to cellular reprogramming

Over the last decade, cellular reprogramming has revolutionized our understanding of the malleability of cell fate. In 2007, Yamanaka and Thomson [16, 111] generated human induced pluripotent stem cells (iPSCs), heralding a new paradigm in cellular programming with significant implications for disease modeling and regenerative medicine. Reprogramming of commonly available cells (e.g. skin and blood cells) into rare, difficult-to-isolate cell types massively advances our ability to model diseases *in vitro*. In this review, we utilize the term ‘reprogramming’ to indicate the conversion of cellular identify from one cell fate to another. While the term reprogramming has historically specified conversion to iPSCs, the process and terminology extend beyond stem cell biology to more generally describe cell-fate conversion. Beyond pluripotent cells, recent studies have shown that transcription factor-mediated reprogramming can convert skin fibroblasts directly into many somatic cell types including neurons, neural precursors, cardiomyocytes, and hematopoietic cells, skipping the time-consuming generation of iPSCs [1–5]. Reprogramming from one somatic cell type to another is often described as transdifferentiation or direct conversion. Although the protocols for generating cell types are as diverse as the cell types themselves, the processes and strategies of reprogramming to pluripotent and somatic cells are highly similar.

Reprogrammed cells provide patient-specific models of complex disease. For diseases and developmental disorders, a constellation of factors (e.g. genetic background, environmental exposure, history of infection) influences the onset and course of disease. By expanding the range of genetic backgrounds to include any donor (e.g. patient-specific), reprogrammed cells fundamentally alter the possibilities for modeling diseases at the cellular level [6]. Relevant disease phenotypes have already been captured in reprogrammed cells, suggesting the potential to use these cells as a tool to study neurological, muscular, and cardiac diseases [7–12].

Beyond modeling disease, reprogramming provides a testbed for studying cellular plasticity. The malleability required for cellular reprogramming poses questions regarding how stable cellular identities are maintained and why certain cells possess differential ability to reprogram or transform. Simple overexpression of four transcription factors redirects the entire trajectory of cellular fate to that of an iPSC. If this relatively simple expression system can direct a fibroblast to become an iPSC, what could we do with more complex synthetic circuitry such as feedback controllers [13]? Reprogramming provides a rich landscape for investigating the performance and potential of synthetic genetic systems to control cell fate.

Providing regenerative therapies through cellular replacements or *in vivo* reprogramming remains one of the most ambitious aims of cellular reprogramming. Over the coming decades, with the development of improved understanding and tools, cellular reprogramming will transform from an *in vitro* science into a translational therapy. However, there remain several challenges to achieving the potential of reprogramming. In this review, we present how various synthetic biology approaches have been used to address challenges and provide a perspective on how recently developed synthetic biology tools and systems biology insights can be applied to further develop the enormous potential of reprogramming. As we gain improved control of the reprogramming process through optimized vectors and tailored protocols, reprogramming will support regenerative medicine by enabling the replacement and repair of damaged tissues.

## Synthetic biology approaches to challenges in cellular reprogramming

While reprogrammed cells hold great potential, low reprogramming rates and immaturity limit the practical use of reprogrammed cells [14,15]. As molecular barriers to efficient reprogramming and maturation have been identified, synthetic biology tools to overcome these barriers have in turn been developed.

### Addressing challenges in delivery and transgene expression

Effective reprogramming requires efficient delivery of reprogramming factors and other genetic elements. Initial reprogramming technologies used retroviruses to infect difficult-to-transfect primary cells [16,17]. However, retroviruses introduce issues with silencing, immunogenicity, and genomic integration [18,19]. As an alternative, a range of new delivery modes have been developed, including nonintegrating viruses and mRNA of reprogramming factors. Each delivery vehicle presents different opportunities and limitations in disease modeling and regenerative medicine.

**Figure 1:**
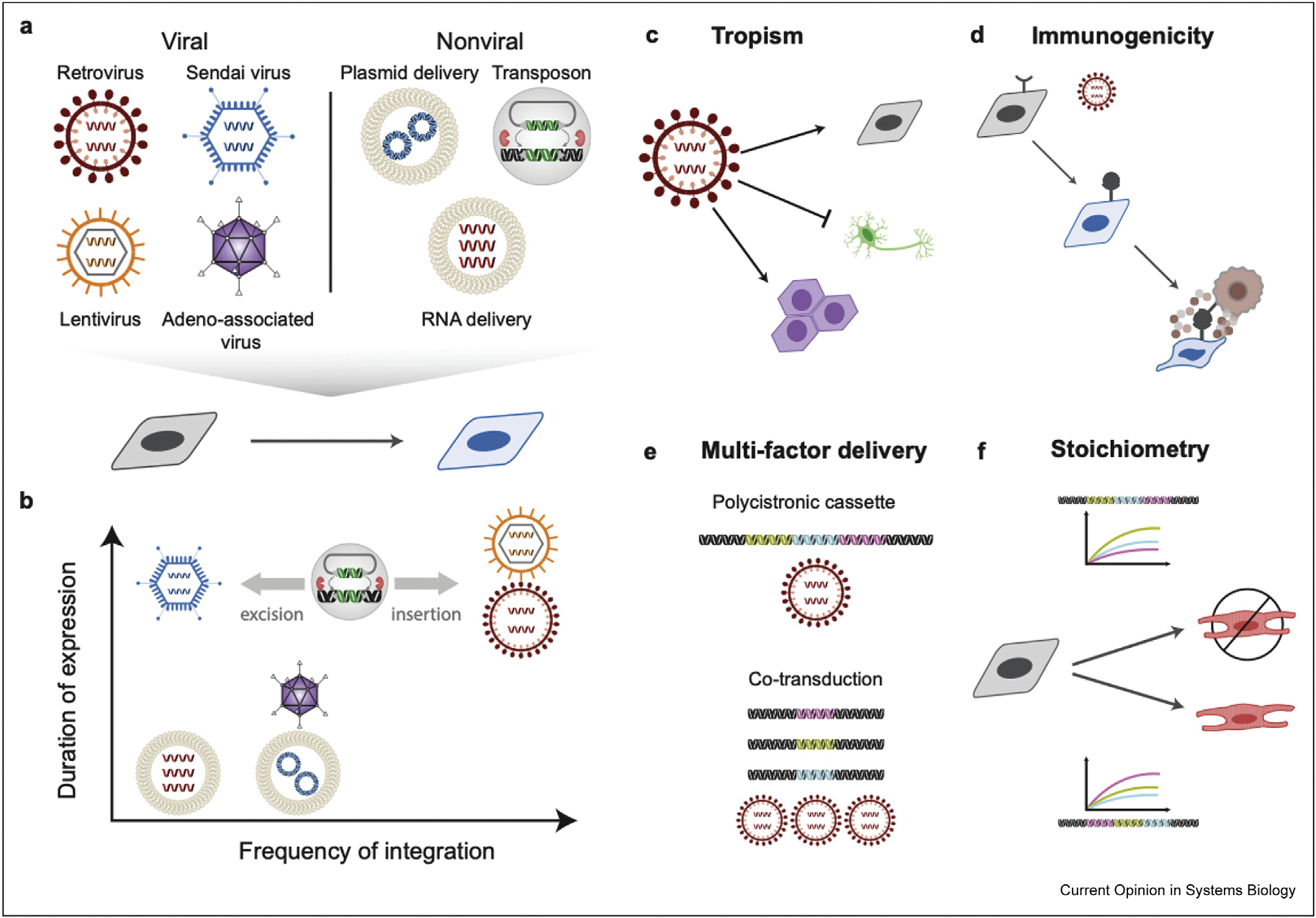
Delivery vectors and transgene expression. **(a)** Viral and nonviral approaches to delivery of reprogramming factors. **(b)** Delivery vectors vary in the duration of expression and the frequency of integration. Retroviruses and lentiviruses integrate with high frequency, enabling sustained expression. Adeno-associated viruses (AAVs) and plasmids integrate on rare occasions and dilute through cell division, resulting in shorter expression duration. In contrast, RNA-based methods and Sendai viruses (SeVs) pose no risk of integration. SeVs provide long-term expression without integration by replicating in the cytoplasm. SeVs can be removed at specific timepoints by different methods, such as siRNA targeting Sendai polymerases [24]. **(c)** Viral delivery methods vary tropism which is their ability to infect various cells types (e.g. dividing vs. nondividing cells, lineage-specific) as well as which area of the body to target. Efficient *in vivo* reprogramming requires delivery targeted to the desired reprogramming site and cell type. **(d)** Viral vectors vary in their immunogenicity. Newer viral delivery methods such as AAVs and SeVs are less immunogenic than conventional retroviruses or lentiviruses [20,23]. **(e)** For multi-factor delivery, polycistronic cassettes ensure the delivery of all reprogramming factors to transduced cells; however, the limited cargo size of AAVs requires separation of factors and cotransduction to achieve delivery. **(f)** The ordering of reprogramming factors on polycistronic cassettes impacts the expression of each factor, resulting in highest expression of upstream genes [33].

Genetic elements that induce reprogramming can be delivered via viral or nonviral vectors (Figure 1a). Viral vectors enable high transduction efficiency, robust transgene expression, and broad tropism (i.e. ability to infect a particular cell type) in primary cells (Figure 1b and c) [20]. Conventional lentivirus- and retrovirus-mediated transductions integrate reprogramming factors into the genome, enabling long-term expression (Figure 1b). However, integration creates the risk of oncogenesis because of random insertional mutagenesis [21]. Integrated transgenes can be eliminated via the Cre-lox recombination system by flanking reprogramming transgenes with loxP sites [22]. Alternatively, nonintegrating viruses such as Sendai viruses (SeVs) and adeno-associated viruses (AAVs) provide better safety profiles while maintaining robust, temporary expression of synthetic constructs. SeVs have been successfully used for *in vivo* reprogramming to replace damaged cardiac tissue in mice [23]. Unlike AAVs, SeVs can provide long-term expression and can be removed at specific timepoints by various methods (e.g. siRNA) [24]. AAVs are widely used for gene therapies because of their low immunogenicity (Figure 1d) [20]. The high cotransduction efficiency of AAVs enables the separation of factors onto individual AAVs which reduces cargo size while still inducing reprogramming *in vivo* [25]. However, AAVs have a low packaging capacity, preventing the delivery of larger multigene cassettes [20].

Nonviral delivery methods of reprogramming factors typically use cationic polymers or lipids/liposomes to directly deliver DNA or RNA into cells [21]. Although rare, delivered DNA can integrate into host genomes (Figure 1b) [26]. To address this issue, nonplasmid-based methods have been developed which pose no risk of integration. Synthetic modified mRNA cocktails can reprogram human primary fibroblasts, sometimes with higher efficiency than viral-mediated methods [27]. While most reprogramming methods rely on overexpression of reprogramming factors, knockdown of a key splicing factor via antisense oligonucleotides (ASOs) converts astrocytes to neurons [28]. In addition, injection of antisense oligonucleotides has proven clinical efficacy and safety in treating neurodegenerative diseases, presenting an alternative delivery method for reprogramming [7,29]. Another nonviral delivery method uses transposon systems, such as PiggyBac or Sleeping Beauty, to temporarily integrate transposons containing reprogramming factors [30,31]. Transposon systems are delivered via plasmids and can be excised when the appropriate transposase is expressed.

Although systemic analyses of these methods give some insight into method selection, there is no comprehensive study of all delivery methods that assesses reprogramming efficiency and maturity of derived cells. Of the semicomprehensive studies, a comparative analysis of viral- and nonviral-mediated delivery methods in reprogramming demonstrated that viral-derived human iPSCs (hiPSCs) have the most similar transcriptome to human embryonic stem cells (hESCs) [26]. Achieving a more complete pluripotent state may require sustained transgene expression enabled by viral delivery [26,32]. However, neither viral- nor nonviral-derived human iPSCs show a difference in cardiac differentiation potential [26]. Together, these observations suggest that delivery methods affect the pluripotent state of reprogrammed cells without compromising differentiation potential. More robust studies will illuminate the advantages and disadvantages of different delivery methods for reprogramming.

Given the importance of delivery, mechanisms enabling multi-factor infection and stoichiometric balancing may enhance reprogramming. Polycistronic cassettes of transcription factor cocktails ensure that transduced or transfected cells receive all transcription factors required for reprogramming (Figure 1e) [4,33,34]. The ordering of transcription factors on the cassette controls transcription factor stoichiometry, resulting in highest expression of upstream genes (Figure 1f) [33]. In cardiomyocyte and dendritic cell reprogramming, cassette ordering impacts stoichiometry and reprogramming efficiency [4,33]. In contrast, during neural reprogramming, widely varying ratios result in successfully induced motor neurons [35]. Given these data, how do we understand the general impact of stoichiometry on reprogramming? One experiment from cardiomyocyte reprogramming may explain these paradoxical observations [23]. Reprogramming was optimized in a retroviral cassette ordering reprogramming factors as Mef2c, Gata4, and Tbx5 (MGT). Surprisingly, rearranging the factors in a SeV-delivered GMT ordering induced cardiomyocyte reprogramming more efficiently than the stoichiometry optimized retroviral MGT. Putatively, the observed reprogramming efficiency increase is because SeV-GMT resulted in higher expression levels of all three reprogramming factors (M, G, and T) compared with retroviral-delivered MGT. Thus, successful reprogramming may require specific transcription factors to exceed certain thresholds rather than meet precise ratios of reprogramming factors.

Tools from synthetic biology may resolve these potentially contrasting observations by systematically perturbing reprogramming factor expression levels. Synthetic promoters operating with orthogonal transcription factors offer predictable expression over a wide range of expression levels [36]. In addition, degradation peptide tags offer predictable control over protein degradation rates [37]. Incorporation of these synthetic regulators into larger gene circuits with feedback control can reduce expression noise across cells [38]. By providing layers of gene expression regulation, synthetic biology will enable precise control over reprogramming factor levels.

### Addressing epigenetic roadblocks to reprogramming

The central hypothesis of transcription factor-mediated reprogramming relies on optimal cocktails of factors redirecting transcriptional networks and, thus, cellular identity. However, epigenetic roadblocks may impede transcription factor-mediated changes by inhibiting access to various loci and prohibiting the activation of critical subnetworks of genes [5,39]. To address this issue, various cocktails of small molecules and genes have been developed to increase genomic accessibility [40–42]. Inhibitors of histone deacetylase block the removal of acetyl groups from histones. These acetyl groups prevent tight DNA-histone binding, putatively enabling higher rates of transcription factor binding to cognate sites [43]. In addition to cocktails to increase DNA accessibility, synthetic biology approaches to overcome epigenetic roadblocks have focused on improving the efficacy of transcription factors to open one or many loci and enable activation of native regulatory networks.

Transcription factors drive changes in chromatin structure by binding to cognate DNA sequences, recruiting transcriptional machinery, and inducing transcription [44,45]. Efforts to drive cellular transitions have focused on transcription factor cocktails to induce specific cell fates [46]. Induction of transcription remodels chromatin, putatively removing epigenetic roadblocks through nucleosome eviction. Synthetic biology efforts to overcome epigenetic barriers have largely focused on engineering more potent synthetic transcription factors.

Synthetic transcription factors such as CRISPR activators (CRISPRa) promote changes in chromatin structure by recruiting transcriptional machinery to induce transcription and epigenetic remodeling. Targeting of just a single locus via CRISPRa can drive large-scale chromatin remodeling needed for cellular reprogramming. CRISPRa targeting *Oct4* or *Sox2* eliminates the need for Oct4 or Sox2 overexpression, respectively, in reprogramming fibroblasts to iPSCs [47,48]. CRISPRa can replace native factors to generate neurons, skeletal muscle, and cardiac progenitors [49–51]. In addition, recent techniques to connect CRISPRa to signaling activity of native pathways provide the potential for signaling-dependent locus activation to enable coordination between signaling activity and reprogramming [52]. While CRISPRa techniques provide programmable site-specific activation, the large size of dCas9 (4.5 kb gene) limits adoption into viral vectors with smaller cargo limits (e.g. AAV). Smaller Cas variants may enable improved flexibility of CRISPRa to target primary cell types [53,54].

Expanding beyond native proteins, library-based approaches enable the selection of novel transcription factors. Evolution of Sox17 via directed evolution of reprogramming factors by cell selection and sequencing identified variants that replaced Sox2 and increased the rate of reprogramming to iPSCs [55]. Selection of zinc finger libraries fused to VP64 identified artificial transcription factor variants that could replace Oct4 overexpression [56]. Effective artificial transcription factors did not directly target Oct4, but instead appeared to activate processes that indirectly induced endogenous pluripotency networks.

Curiously, exclusion of Oct4 from Klf4, Sox2, and cMyc (KSM) cocktails modestly reduces efficiency but improves the developmental potential of iPSCs, increasing the rate of live-born chimeric mice [32]. Together these data suggest that identifying optimal reprogramming protocols require defined objectives whether efficiency, maturity, or potential. As greater insight into the central processes that support reprogramming emerge, the range of useful synthetic transcription factors and combinations will continue to expand via directed evolution.

### Overcoming challenges from latent donor identity

**Figure 2:**
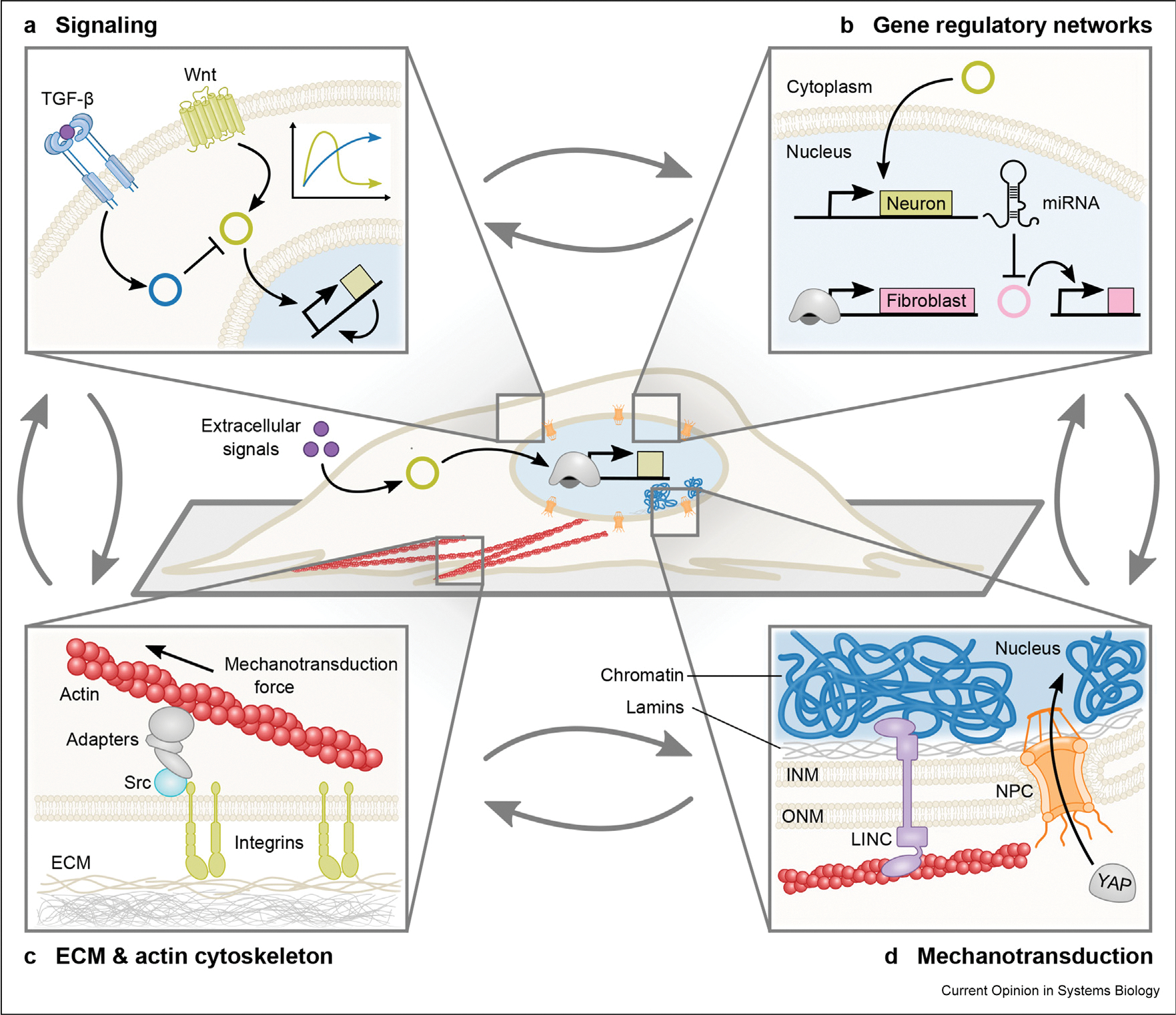
Challenges in latent donor cell identity. Residual expression of the donor gene regulatory networks (GRNs), vestiges of the cytoskeleton, and other biomolecules defines the latent identity of donor cells. Through interconnection of properties, latent donor identity affects the efficiency and maturity of reprogrammed cells. **(a)** Inhibition or activation of signaling pathways may increase or impede reprogramming, depending on the maturity of the donor cell and reprogramming protocol. **(b)** Donor GRNs can be repressed by microRNA, allowing cells to more readily adopt new cellular identities. **(c)** Mechanical cues from the ECM are transmitted through the actin cytoskeleton and focal adhesions which are composed of integrins, Src kinases and other adaptor proteins. Cells respond to mechanical cues via mechanotransduction (Figure 2d). **(d)** Mechanotransduction directly relays mechanical cues to the nucleus via the linker of nucleoskeleton and cytoskeleton (LINC) complex [108]. Mechanotransduction can also transmit secondary signals via transcription factors such as YAP, which translocates into the nucleus via nuclear pore complexes (NPCs) in response to mechanical forces. ECM, extracellular matrix; miRNA, microRNA; YAP, Yes-associated protein; INM, inner nuclear membrane; ONM, outer nuclear membrane.

Competition between established and newly induced gene regulatory networks (GRNs) to define central cellular properties (e.g. the actin cytoskeleton, splicing, the ensemble of secreted extracellular matrix [ECM] proteins) may limit the full adoption of the alternative identity during reprogramming, resulting in a spectrum of conversion. Latent activity of donor cell GRNs impacts direct conversion and may compromise cellular maturity [57]. Given their interconnection, perturbations of native signaling pathways, the cytoskeleton, and the ECM provide nodes for actuating changes in GRNs to improve the efficiency and maturity of reprogrammed cells (Figure 2).

Native signaling pathways in donor cells provide diverse levers for tuning reprogramming (Figure 2a). For example, addition of small-molecule inhibitors of pro-inflammatory transforming growth factor (TGF)-β signaling can improve reprogramming efficiency [58–61]. TGF-β signaling may impede reprogramming by inducing fibrosis and senescence [62]. In addition, inhibition of the inflammatory cytokine interleukin (IL)-1β and its downstream effectors promotes reprogramming to cardiomyocytes in adult mouse fibroblasts [63]. Inhibition of this pro-inflammatory cascade improves reprogramming of post-natal and adult fibroblasts but has no effect on embryonic fibroblasts. The existence of divergent inflammation responses based on developmental stage suggests that additional layers of regulation via native signaling can prevent reprogramming of mature cell types. Senescent cells secrete IL-6 which augments the generation of iPSCs non–cell-autonomously (i.e. through multicellular interactions) [64,65]. Putatively, the IL-6/STAT3 signaling pathway induces expression of the transcription factor NKX3–1, which activates expression of endogenous Oct4 [66]. The impact of Wnt signaling on reprogramming is highly context-dependent. Activation of the Wnt signaling pathway increases reprogramming of fibroblasts to iPSCs [61]. In addition, Wnt signaling triggers proliferation and conversion of Müller glial cells to neurons [67]. However, Wnt inhibition promotes reprogramming to cardiomyocytes [63]. Given the diverse, cell-specific functions of native signaling pathways, connecting signaling to mechanisms of reprogramming remains an important objective. Moreover, tools for connecting signaling to regulation may enable genetic control that is tuned to pathway activity. There exists enormous potential for synthetic biology approaches to modulate and redirect these pathways (e.g. generating pathway-responsive transcription factors [52]), providing selective, responsive, single-cell control of reprogramming within heterogenous cell populations. The potential of these strategies is discussed in further detail in section A vision for the future of synthetic biology in reprogramming.

Native signaling pathways, the epigenetic state, and cellular identity all connect with the cytoskeleton and ECM by mechanotransduction [68]. In addition to providing mechanical support, the cytoskeleton enables cells to sense and respond to changes in their environment’s mechanical properties through mechanotransduction (Figure 2c and d). Furthermore, stages of reprogramming correlate with changes in cell stiffness [69]. Emerging studies demonstrate that leveraging mechanical cues to affect cellular changes may enhance reprogramming efficiency and cellular maturity.

Mechanical properties of substrates affect both directed differentiation and iPSC reprogramming. For example, substrate stiffness can interact with soluble induction factors to direct differentiation and dictate lineage commitment of PSCs/iPSCs [70,71]. Current methods to improve reprogramming by modulating cell stiffness either target the actin cytoskeleton or change the substrate’s mechanical properties. Softer substrates result in less stiff actin fibers and facilitate reprogramming of mesenchymal stem cells into iPSCs [72]. Recent evidence demonstrates that cell stiffness can block chromatin accessibility and full pluripotency [69]. Mechanotransduction signaling can also influence reprogramming non–cell-autonomously. For instance, overexpressing the Yes-associated protein (YAP), a mechanosensitive transcriptional co-activator, increases reprogramming of co-cultured cells but does not increase reprogramming of YAP-expressing cells [73]. The complex interplay of signaling and mechanical cues and their feedback into GRNs necessitates an integrated approach to modeling and probing pathways and processes in cellular fate transitions.

Given the highly intertwined cellular processes and competition to define cellular properties, repressing competing GRNs via microRNAs (miRNAs) represents an important tool in reprogramming (Figure 2b). Overexpression of cell type-specific miRNAs alone or in combination with transcription factors enables reprogramming to iPSCs [27], cardiomyocytes [74], and neurons [75]. miRNAs alleviate the barriers to reprogramming by directly repressing donor cell GRNs and derepressing the target cell GRNs. In reprogramming fibroblasts to cardiomyocytes, supplementing the cardiomyocyte transcription factor cocktail with miR-133 has been reported to directly repress the fibroblast transcription factor Snai1 [76]. In neuronal reprogramming, miR-9/9*-124 expression depletes restrictive element-1 silencing transcription (REST) [77]. REST represses expression of neuronal genes in non-neuronal cells. miRNA-mediated depletion of REST increases chromatin accessibility at REST-binding sites to generate neurons primed for subtype specification [78,79]. Beyond influencing DNA-binding proteins and chromatin architecture, the feedback between miRNAs and RNA-binding proteins regulates cell identity. In non-neuronal cells, polypyrimidine tract binding (PTB) binds to RNAs targeted by miR-124, blocking the binding of miR-124. In the absence of miR-124–mediated targeting and downregulation, expression of REST and other non-neuronal factors increase, repressing expression of miR-124 [80]. By exploiting this circuit architecture, knockdown of PTB induces expression of miR-124 to convert astrocytes to neurons *in vivo* [28]. miRNAs may also exhibit a synergistic effect with RNA-binding proteins to promote conversion [81]. Through post-transcriptional and downstream post-translational effects, miRNA expression directly influences the epigenetic barriers and donor-cell GRNs to promote reprogramming.

The combination of signaling pathways, GRNs, epigenetic state, and cytoskeletal elements defines the molecular determinants of cellular identity that confer unique cellular forms and functions. Whereas single molecular determinants may coordinate individual processes, multiple layers of regulation support the maintenance of stable identities, buffering cells against cell-fate transitions. The highly integrated nature of these molecular networks requires a systems-level approach and precise tools to dissect the multiple regulatory layers that reinforce cellular identity.

## From tools to systems: insights from the systems biology of reprogramming

**Figure 3:**
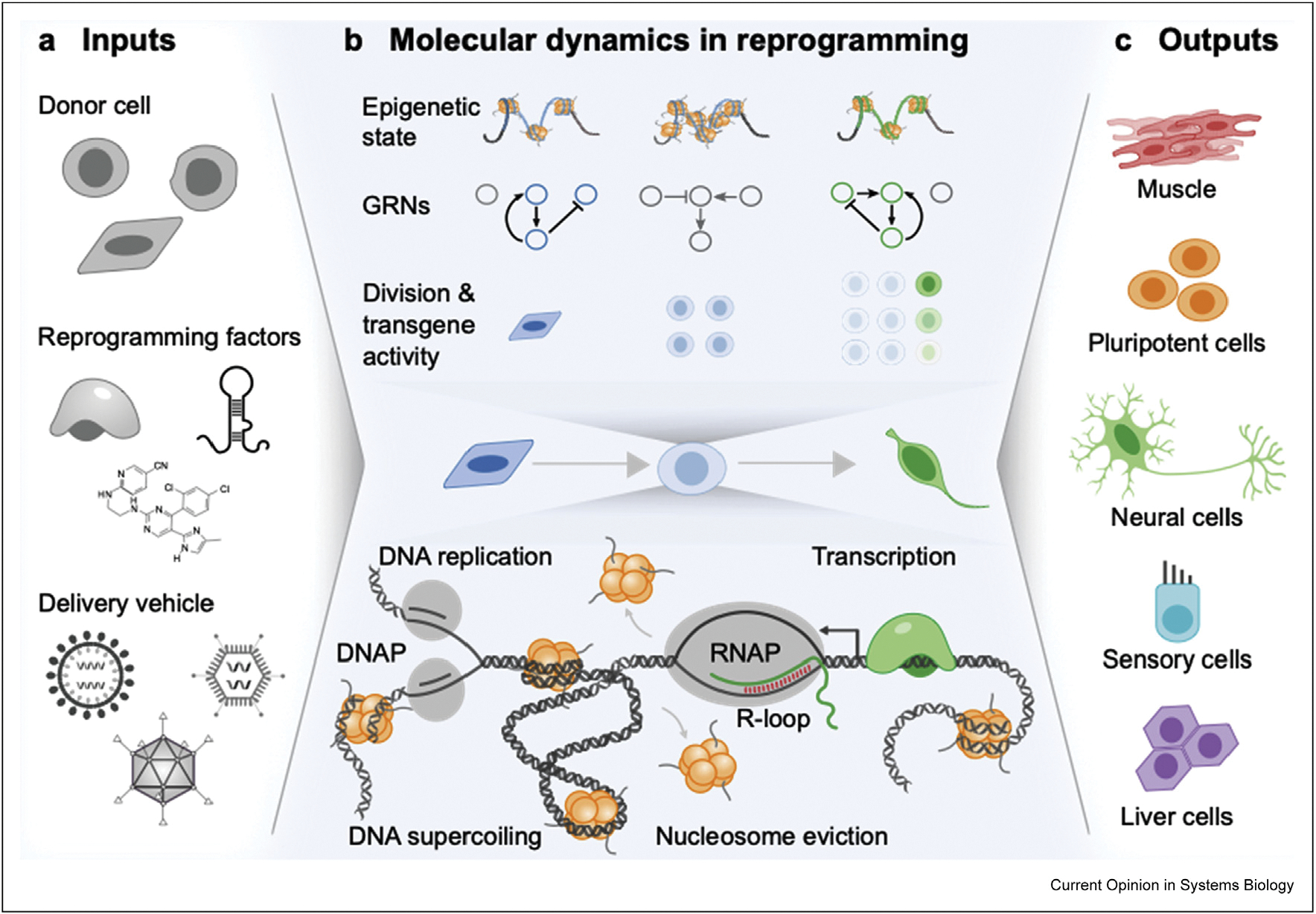
A range of inputs induce molecular dynamics to guide cells to their reprogrammed state. Cellular reprogramming requires dynamic integration across multiple scales from biomolecules to gene regulatory networks (GRNs). **(a)** To induce reprogramming, reprogramming factors including transcription factors, microRNAs, and small molecules are introduced to donor cells through a range of delivery methods. **(b)** The process of reprogramming induces epigenetic remodeling to close donor loci and silence donor GRNs, while new loci open and GRNs for the alternate identity activate. Cell division promotes reprogramming and may facilitate dilution of latent donor identity [109]. Sustained transgene activity in hyperproliferative cells is limited, but rare hyperproliferative cells capable of sustaining transgene activity reprogram efficiently [58]. At the molecular level, high rates of transcription and proliferation induce genomic stress during reprogramming by increasing in supercoiling, R-loop formation, and polymerase collisions. Transcription generates forces capable of evicting nucleosomes, enabling some epigenetic remodeling. **(c)** Cells capable of mitigating genomic sources of stress reprogram at near-deterministic rates into a broad range of cells. DNAP, DNA polymerase; RNAP, RNA polymerase.

Operating on the paradigm that reprogramming is limited by delivery modes, latent donor identity, and the epigenetic state, synthetic biology efforts have focused on developing tools to overcome these barriers. Going forward, engineered systems-level tools will be needed to robustly regulate proliferation, maintain transgene activity, and facilitate chromatin rewiring (Figure 3). Much as the first wave of synthetic biology pioneered tools in bacteria that paved the way for sophisticated systems, the next wave of synthetic biology in reprogramming will transition from tools to systems.

The rarity of reprogramming suggests that ‘privileged’ populations exist and preferentially reprogram. Lineage tracing experiments confirm that elite clones dominate successful reprogramming events [82]. Recently, mechanisms that mark privileged cells have been identified [58,83]. Fast-cycling cells preferentially reprogram into iPSCs [83]. Beyond iPSCs, hyperproliferation promotes reprogramming into multiple, post-mitotic lineages, suggesting transient hyperproliferation broadly enhances reprogramming [58]. In the context of conversion, rapid replication may facilitate dilution of highly stable mRNAs, miRNAs, and proteins that may otherwise limit full adoption of an alternative identity. Designing reprogramming protocols to facilitate the dilution of molecular components may improve reprogramming efficiency and maturity of the derived cells.

Maintaining transgene expression in hyperproliferative cells may present a challenge for improving reprogramming induced via retroviral delivery. Silencing of transgene expression from retroviruses occurs at a greater frequency in hyperproliferative cells [58]. In addition, retroviral silencing appears to be dependent on the presence of particular sets of reprogramming factors and may occur independently of reprogramming [32]. For instance, Sox2 and Myc coexpression activates retroviral-silencing machinery in somatic cells before induction of key markers of pluripotency [32]. Somewhat paradoxically, retroviral silencing in cells expressing a fluorescent marker correlates with higher rates of reprogramming [84]. However, retroviral-delivered transgene silencing (e.g. loss of marker expression) may simply correlate with proliferation rather than serve as a sign of successful reprogramming. We have recently demonstrated that sustained transgene activity distinguishes complete from partial reprogramming [58]. Thus proliferation-mediated processes may generate a tradeoff in factors influencing reprogramming by promoting higher rates of reprogramming while silencing transgene expression. Based on our findings, we expect that proliferation promotes loss of latent donor identity and simultaneously induces cell cycle–mediated transgene silencing. Expanding the population of hyperproliferative cells capable of sustaining transgene activity increases reprogramming efficiency and maturity of derived cells [58]. Although transgene silencing is well-established for retroviruses, it remains unclear whether other delivery mechanisms are limited by cell cycle-mediated silencing or could eliminate tradeoffs between proliferation and transgene expression.

Beyond hyperproliferation and transgene expression, nuclear organization and differential rates of transcription have been identified as important markers of reprogramming potential [44,45,58,85,86]. Changes in the structure around noncoding regulatory regions such as enhancers may facilitate changes in nuclear structure and gene expression that promote reprogramming [44,86]. In addition, transcription drives changes in chromatin structure and nuclear organization [87,88]. Thus, higher transcription rates may facilitate more rapid chromatin remodeling and induction of nuclear organization that facilitates the establishment of new GRNs. As yet, it remains unclear whether the process or the products of high rates of transcription enhance reprogramming.

## A vision for the future of synthetic biology in reprogramming

Enabling precise, temporal control of gene expression via gene circuits is a hallmark of synthetic biology. High-gain feedback controllers may enable precise control of transcription factor expression [89]. While the theory and tools to engineer genetic controllers have rapidly expanded in mammalian systems, the key control objectives in reprogramming remained poorly defined until recently. Our recent work illuminates opportunities for tailoring transcription factor expression within asynchronously reprogramming cells through dynamic objectives [58].

**Figure 4:**
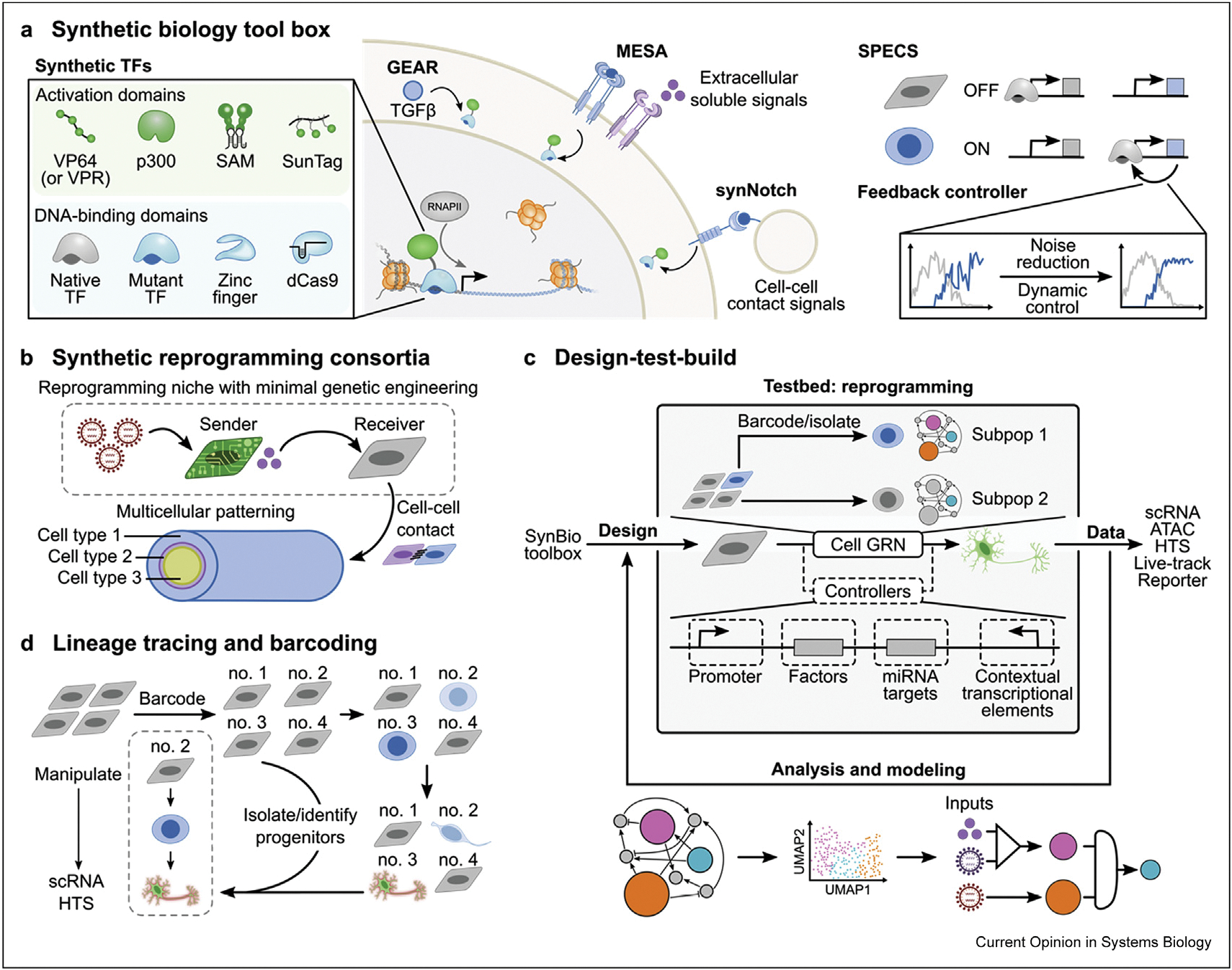
Future perspectives on the synthetic biology of reprogramming. **(a)** Newly developed synthetic biology tools will enable more sophisticated control over cells during reprogramming. Synthetic molecular sensor devices such as the signaling pathway-responsive generalized engineered activation regulators (GEAR) [52], or the ligand-sensitive receptors modular extracellular sensor architecture [97] and synthetic notch receptors (synNotch) [96], transmit intracellular signaling and extracellular binding events, respectively, into release of transcription factors. Sensor devices are important tools in developing genetic feedback controllers. Molecular sensor devices may be coupled with synthetic transcription factors which are composed of two domains: a DNA-binding domain (confers sequence specificity) and an activation domain (induces site-specific induction of gene expression) [46–49,55,56,110]. In addition, transcriptional control through cell state-responsive promoters such as synthetic promoters with enhanced cell-state specificity (SPECS) profile a facile mechanism for composing feedback controllers that can be layered to enable precise and dynamic control [90,91,112]. **(b)** Future reprogramming strategies will exploit non-cellSynthetic Biologyautonomous effects to facilitate cell-fate transitions by constructing multicellular systems that interact synergistically to enhance reprogramming. Notably, oncogenic circuits could be engineered in ‘sender’ cells that promote reprogramming in minimally genetically modified ‘receiver’ cells via intercellular signaling [64,65,73,95]. Furthermore, cell-cell contact signaling responsive tools (e.g. synNotch) can be integrated to build self-patterning multicellular tissues during reprogramming [96]. **(c)** Synthetic biology provides a host of tools to control gene regulatory networks during reprogramming. By following a design-test-build strategy, synthetic circuits may enable the identification and targeting of dynamic objectives during reprogramming [58]. **(d)** Recently developed lineage tracing and barcoding tools will be essential in identifying traits and events that allow cells to reprogram efficiently by combining longitudinal live tracking with next generation sequencing methods, such as single-cell RNA sequencing (scRNA) [99,104].

While transcription drives changes in the cellular state, high transcription rates early in reprogramming inhibit proliferation and stall reprogramming [58]. Consequently, hypertranscription and hyperproliferation represent dynamic objectives. Developing strategies to reduce transcription from donor GRNs and tune expression of reprogramming factors may facilitate more rapid, efficient reprogramming. To effectively balance both processes, reprogramming vectors tailored to scale transcription with the capacity of individual cells may improve reprogramming strategies by limiting transcriptional strain on the genome. Simple selection of promoters to regulate transcription factor expression may be sufficient to improve expression scaling from transgenic constructs (Figure 4a). Recently developed libraries of cell state-specific promoters offer a diverse array of elements for tuning gene expression [90]. In addition, given the role of signaling pathways to induce cell-fate changes, signaling-responsive genetic controllers may enhance reprogramming by coordinating signaling, the cell cycle, and transcription factor expression [52]. Alternatively, more complex feedback control systems such as bandpass filters and pulse generators may provide temporally-defined pulses of gene expression [91,92]. Linking these regulatory tools together would generate controllers capable of sensing cell state and coordinating the optimal actuated responses that guide each cell to its new identity.

While reprogramming has primarily focused on cell-autonomous engineering, non–cell-autonomous interactions contribute to a range of cellular behaviors [73,93,94]. Development of synthetic multicellular interactions may facilitate precise cell-cell communication for sophisticated spatiotemporal coordination and enable complex maturation processes. For example, YAP-expressing cells increase the reprogramming rate of YAP-negative cells via intercellular signaling [73]. Similarly, IL-6 secretion augments the generation of iPSCs non–cell-autonomously [64,65]. Non–cell-autonomous processes represent opportunities to segregate engineered systems into well-defined ‘sender’ cells and ‘receiver’ cells (Figure 4b). Developing multicellular reprogramming niches composed of sender and receiver cells may enable coordination of multicellular processes as well as restrict specific genetic manipulations to one subset of cells. For example, overexpression of mutant RAS induces proliferation in a non-cell-autonomous manner [95]. Given proliferation is a key determinant of reprogramming, oncogenic programs that promote proliferation can be restricted to sender cells, preserving an oncogene-free profile for reprogrammed receiver cells. The development of synthetic reprogramming consortia may accelerate the translation of reprogrammed cells by enhancing reprogramming efficiency with limited genetic manipulation.

Beyond the development of synthetic reprogramming consortia, complex patterning may be facilitated by synthetic receptor systems to enable spatiotemporal control of cell fate [96]. Synthetic receptor systems provide an orthogonal mechanism by which to coordinate expression of reprogramming factors with extracellular signaling [96–98]. For example, modular extracellular sensor architecture (MESA) receptors respond to soluble extracellular signals by releasing a tethered transcription factor to induce gene expression [97]. Similarly, synthetic Notch receptors (synNotch) release tethered transcription factors in response to cell–cell contacts [96]. By engineering synNotch-controlled expression of cadherins, cells of varying adhesivity can be programmed to form self-patterning multicellular structures [96]. Combining integrated multicellular consortium approaches with synthetic receptor systems that regulate diverse cells fates will provide a unique context to study and build multicellular tissues.

Circuits represent small-scale transcriptional networks that can be used to model large-scale networks. The impact of these ‘small-scale’ systems to label and identify unique populations and cellular states will be magnified by connection to ‘large-scale’ system biology techniques including genomics, epigenomics, and genome architecture. While large-scale systems provide high-dimensional profiling at single timepoints, small-scale systems such as circuits enable dynamic tracking from low-dimensional, integrated metrics such as circuit activity. By connecting experimental designs across these scales, we can enhance the ‘design-build-test” workflow to develop live-cell reporters of unique cell states, to optimize genetic controllers, and to develop strategies for assessing and enhancing the maturity of reprogrammed cells (Figure 4c).

Lineage tracing tools provide a method for examining how individual clones contribute to reprogramming (Figure 4d). Given the rarity of reprogramming and the heterogeneous nature of cellular responses, synthetic biology approaches to reconstruct reprogramming lineages and isolate progenitors of successfully reprogrammed cells may clarify the role of donor cell-intrinsic barriers to reprogramming. Lineage tracing via uniquely barcoded starting cell populations has identified that a relatively small set of elite clones reprogram from MEFs to iPSCs [82]. Recent methods for retrospectively identifying clones expand the potential of barcoding. The CRISPRa tracing of clones in heterogeneous cell populations (CaTCH) system relies on split populations of cells labeled with a barcode upstream of a reporter gene. Clones selected by phenotype are enriched and identified by sequencing. Introduction of a guide RNA to the unique barcode of the enriched clone activates reporter expression, enabling live tracking of the clone through various perturbations [99]. With this method, it is possible to ask questions about clonal determinism and ergodicity in cell-fate transitions.

Building on these static barcoding strategies, newly developed tools implement dynamic barcoding, allowing not only identification of a progenitor clone, but reconstruction of the entire cell lineage [100,101]. Dynamic barcoding relies on stochastic recombination events via Cas9 or other DNA recombinases to uniquely mark cells during reprogramming [100,101]. To improve tracking, barcodes can be read out *in situ*, preserving spatial and morphological data [100–102]. Importantly, barcodes may also be used in conjunction with single-cell RNA sequencing methods by placing the barcode in the untranslated region of a transgene to map the transcriptional profile to unique cells and populations [103,104]. While lineage tracing requires constitutive action of DNA editing machinery (Cas9, sgRNA), event recording is enabled by linking editing to cellular events such as signaling [100]. Parallel implementation of lineage tracing and event-recording circuits may elucidate transient events that enable or inhibit reprogramming. Furthermore, event-recording circuits that activate live-cell reporters will provide enhanced temporal resolution of subcellular events during longitudinal tracking of reprogramming populations and may also be read using sequencing technologies [105]. Understanding the molecular barriers to various cell states in reprogramming may facilitate strategies to promote maturation.

By connecting lineage and event information with single-cell transcriptomics, synthetic biology enables the connection of ‘small-scale’ events to ‘large-scale’ systems data and may elucidate mechanisms by which elite cells overcome the barriers to reprogramming and maturation (Figure 4c). Incorporating genomics data into the ‘design-build-test’ cycle will significantly improve our understanding and engineering of synthetic genetic controllers of cell-fate transitions.

Although pioneered in single-cell organisms, synthetic biology’s recent expansion to mammalian systems provides a rich landscape of cell fates to examine and engineer [36,52,91,106,107]. As novel tools for studying signaling, chromatin structure, and gene regulation in mammalian systems continue to develop, synthetic biology will enable an unprecedented insight into the mechanism of reprogramming and expand our power to engineer reprogrammed cells.
